# Perinatal/Neonatal case presentation: pulmonary artery sling associated with respiratory distress

**DOI:** 10.1186/s40064-015-1656-5

**Published:** 2016-01-13

**Authors:** David Healey, Nitin Ron, Andrew Hromada, Manoj Chhabra

**Affiliations:** Division of Developmental Medicine, Seattle Children’s Hospital, University of Washington, Seattle, WA USA; Department of Pediatrics, New York Methodist Hospital, 506 Sixth Street, Brooklyn, NY 11215 USA; Weill Cornell Medical College, New York, NY USA; Department of Internal Medicine, Elmhurst Hospital, Queens, NY USA; Icahn School of Medicine at Mt. Sinai, New York, NY USA

**Keywords:** Pulmonary artery sling, Stovepipe trachea, Congenital, Cardiovascular, Neonate, Infant, Developmental origins of health and disease

## Abstract

Pulmonary artery sling is a very rare cause of pediatric respiratory distress. The estimated prevalence of the disease was first determined by Yu et al. in 2008 as 59 per million school-aged children. Associated symptoms are cough, wheezing, and feeding difficulty, all of which are common in routine outpatient pediatric clinical encounters. We report a case of a premature male neonate twin who was admitted to the neonatal intensive care unit with respiratory distress and pneumothorax. His presentation, as well as the etiology of his pulmonary disease, was felt to be consistent with those of numerous other premature infants. Akin to this was his delayed discharge on account of his slow progress with oral feeding. Parents gave a history of tachypnea and feeding difficulty to his doctors. He presented twice to the emergency room in respiratory distress. At 4 months of age, while in hospital for a pulmonary infection, he had an echocardiogram that revealed a pulmonary artery sling. We review the literature on this vascular anomaly, discuss its diagnosis and management, and critique the clinical thinking that determined this child’s course from the perspective of availability heuristics.

## Introduction

Pulmonary artery sling (PAS) is a very rare cause of pediatric pulmonary distress and tracheomalacia. The estimated prevalence of the disease was first determined in 2008 as 59 per million in a large-scale pre-sports participation cardiovascular screening study of 186,213 school-aged children by Yu et al. (2008). All patients identified in the study were unrecognized due to non-specific symptoms of asthmatic cough and recurrent broncho-pulmonary infections. We present here a male child initially admitted to the neonatal intensive care unit (NICU) with respiratory distress who turned out to have a PAS. We also review the literature on PAS and discuss its diagnosis and management, and critique the clinical thinking that determined this child’s course from the perspective of availability heuristics.

PAS is a very rare congenital disorder where the left pulmonary artery (LPA) arises outside the pericardium as a posterior branch off the right pulmonary artery (RPA). It then circles around the distal trachea and right main stem bronchus as it travels between the trachea and esophagus toward the hilum of the left lung. In doing so, it indents these structures and displaces the trachea to the left. The ligamentum arteriosum or the ductus arteriosus originates from the main pulmonary artery (MPA) and passes anteriorly and superiorly to the left main stem bronchus to join the descending thoracic aorta, completing the vascular ring (Fiore et al. 2005). PAS manifests clinically with significant tracheal and esophageal symptoms.

## Case description

A newborn boy was admitted to the NICU for respiratory distress. He was born at 34 weeks gestational age, twin of a dichorionic diamniotic pregnancy, at appropriate weight. His mother was 21 years old, G4P1, with all labs negative, in preterm labor. The other twin was normal. His mother had received regular prenatal care as well as adequate corticosteroids before delivery. Routine resuscitation was given to him. His Apgar scores at 1 and 5 min were 9 and 9. Within minutes, he began to develop significant respiratory distress and had diminished air entry on auscultation.

Two flat and one right lateral decubitus chest X-rays were performed to rule out pneumothorax. They showed a 5.5 cm left pneumothorax as well as hazy densities suggestive of respiratory distress syndrome (RDS). The baby was stable on nasal intermittent positive pressure ventilation (NIPPV) with saturations continuously greater than 90 %. On day 3, the patient was weaned to nasal cannula and subsequently to room air and was stable until discharge. No cardiac abnormalities were evident.

At 2 months of age, the patient presented to the emergency department for cough. Scant wheezes were auscultated. He was diagnosed to have a viral respiratory infection and was discharged home. At 4 months of age, he presented with subcostal retractions and bilateral wheezing from respiratory syncytial virus (RSV) bronchiolitis and was admitted. His mother provided an interim history of persistent tachypnea since birth and feeding difficulty when compared to his twin sister. Radiographs of the chest showed no pleural effusion, pneumothorax, or pulmonary congestion. However, hyperinflation and increased peribronchial markings suggested underlying reactive airway disease, and minimal increased opacity in the right infrahilar region suggested a focal infiltrate or atelectasis. Barium contrast esophagogram was performed to investigate the feeding difficulties. It revealed a subtle contour change in the esophagus above the level of the carina. At this time, a pediatric resident auscultated a systolic murmur at the left upper sternal border, and cardiology was consulted.

**Fig. 1:**
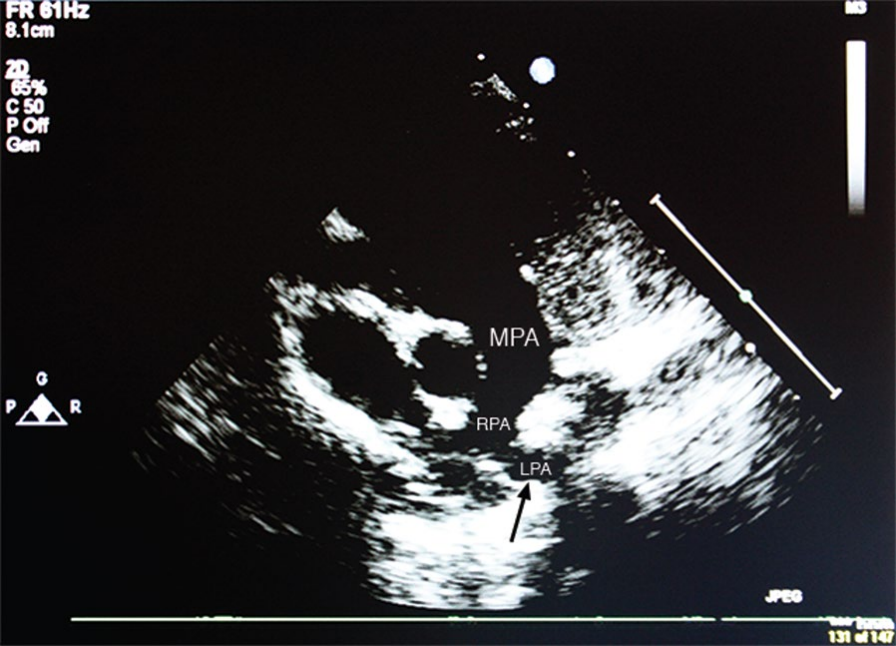
Transthoracic echocardiogram showing main pulmonary artery (*MPA*) giving rise to right pulmonary artery (*RPA*) with arrow depicting left pulmonary artery (*LPA*) arising from its distal segment

**Fig. 2:**
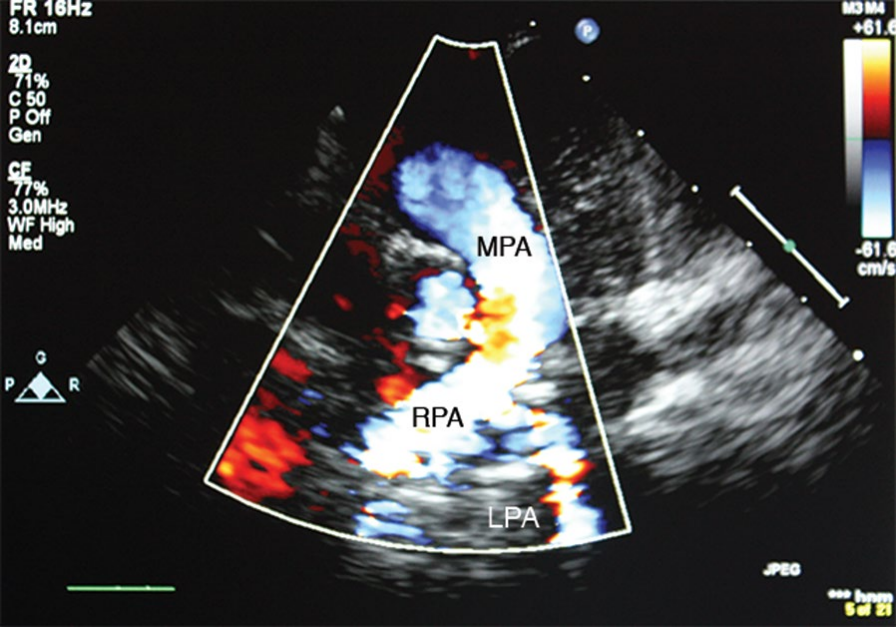
Transthoracic echocardiogram with Doppler showing main pulmonary artery (*MPA*), right pulmonary artery (*RPA*) and left pulmonary artery (*LPA*) flow. Note turbulence within the LPA segment as it courses between the trachea and esophagus

**Fig. 3:**
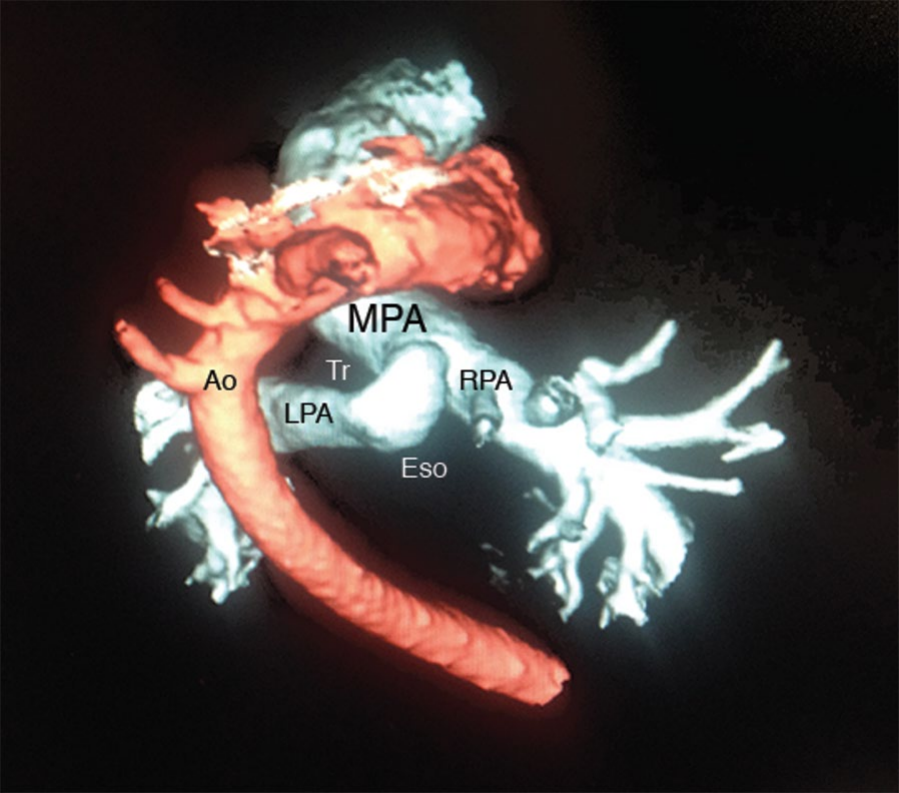
Posterior view of 3D computed tomography angiogram reconstruction showing the main pulmonary artery (*MPA*) giving rise to the left (*LPA*) and right (*RPA*) pulmonary arteries. A pulmonary artery sling is formed as the LPA makes an acute angle encircling the trachea (*Tr*), runs up against the esophagus (*Eso*), and finally passes anterior to the aorta (*Ao*)

Cardiac echo revealed a PAS with the LPA arising from the RPA (Figs. 1, 2). There was good left ventricular function with mild hypoplasia of the LPA. Esophageal fluoroscopy showed a concave contour change in the anterior esophagus just above the level of the carina with no significant reduction in the esophageal lumen size at this level. A bronchoscopy was performed and a 3D chest CT was obtained (Fig. 3).

At 8 months of age, the patient underwent surgical repair of the PAS by re-implantation of the LPA from the MPA without the need of tracheoplasty. He is since faring well.

## Discussion

In this report we describe a premature infant with respiratory distress whose PAS was not diagnosed until 4 months of age. This case highlights the typical presentation of PAS, methods of diagnosis of such a vascular ring, and the importance of including rare diagnoses within the differential.

Current understanding is that PAS has multifactorial causes. Genetic-related factors may play a role, as seen in case reports of PAS in identical twins and in patients with trisomies 18 and 21. A pulmonary artery is formed during prenatal life from two portions; one that derives from the ventral sixth branchial arch, and another that derives from a postbranchial vessel. This latter portion, in turn, arose from the pulmonary capillary plexus that envelops the lung bud. Pu and colleagues hypothesized that if the left postbranchial vessels cannot connect to the left sixth branchial arch, then they might capture a vascular supply from the most nearby major artery. When such a connection is made to the right sixth branchial arch through the embryonic peritracheal mesenchyme between the trachea and the esophagus, a PAS results (Pu et al. 1996).

The first to describe the condition were Glaevecke and Doehle in 1897 (Glaevecke and Doehle 1897). Contro et al. used the term “vascular sling” to distinguish this entity from a vascular ring (Contro 1958). Several other anatomical characteristics describe this disorder. The trachea has complete rings in 50–65 % of cases. Instead of having the normal U shape, the cartilage is circular, hence the term “stovepipe trachea.” In this case, there is no membranous trachea—there is a narrowing of the segment with complete rings. Because of the compression of the lower trachea, patients often present with wheezing and stridor. Atelectasis, air trapping and pneumonia arise from bronchial compression mostly on the right. Sudden death can result in neonates and infants from failure to recognize these symptoms (Sade et al. 1975).

A population of 18 patients was studied by Pawade et al. Among these, symptoms were first noticed between 1 day and 4 months of age. All had wheeze, 11 had additional stridor, 7 had recurrent chest infections and 3 fed poorly and failed to thrive. Predominant signs were stridor, tachypnea, and intercostal retractions (Pawade et al. 1992). Gikonyo et al. reported on 130 cases and found that the ratio of male to female patients was 3:2. Of the 130 patients surveyed, 90 % were symptomatic; 90 % of these were symptomatic in the first year of life, and nearly half of these were symptomatic in the newborn period. When present, symptoms were usually those of respiratory obstruction characterized by stridor and wheezing. The obstruction was maximal during inspiration and there was no associated dysphagia (Gikonyo et al. 1989).

PAS is frequently associated with tracheal anomalies, congenital heart disease, and lung abnormalities. The development of noninvasive imaging modalities such as computed tomography (CT), ultrasonography and magnetic resonance imaging has led to increased reports of this entity. Chen et al. reported the relative frequencies of such anomalies in eighteen cases of LPA sling and used Chi square tests to compare the probability of coexistence of the anomalies. They found that 100 % presented with tracheal stenosis, with a high incidence of combined right tracheal bronchus (22 %), underdeveloped right lung (22 %), persistent left superior vena cava (22 %) and left PDA (39 %). There was a statistically significant correlation of associated anomalies between those with PAS as compared to those without (Chen et al. 2007). Coexisting diffuse tracheal stenosis, creating a ring-sling complex, is identified in up to 65 % of patients with PAS. Typically, stenosis of the trachea is due to complete tracheal rings and ranges from a profound degree of hypoplasia of the entire tracheobronchial tree to a discrete stenosis. Interestingly, despite compression of the tracheobronchial tree by the sling, tracheomalacia is usually not a feature. Congenital heart defects are found in 50 % of PAS cases, the most common being atrial and ventricular septal defects, patent ductus arteriosus, left superior vena cava and Tetralogy of Fallot (Sade et al. 1975).

As described by Hraška et al., treatment of patients with PAS requires close collaboration of pediatric cardiac surgeons and their medical counterparts. Diagnosis of PAS is optimally made using echocardiography, as it is rapid and non-invasive. All infants should undergo bronchoscopy to rule out tracheal stenosis secondary to congenital complete tracheal rings (Hraška et al. 2009).

PAS, once diagnosed, is an indication for surgical repair. Repair using a strategy of median sternotomy, cardiopulmonary bypass (CPB), LPA division and re-implantation into the MPA with simultaneous tracheal repair takes preference. Tracheal repair should be considered only in clinically symptomatic patients. Coexisting intracardiac pathology is repaired or palliated at the same time. The postoperative care of these patients requires close vigilance by pediatric intensivists, anesthesiologists, and pediatric cardiac surgeons. The management of the reconstructed trachea requires close collaboration among these specialties in order to achieve the best long-term result (Hraška et al. 2009; Backer et al. 1999). The clinical outcome of patients with PAS is generally excellent—the outcome depends mostly on other medical issues, such as the associated tracheal lesions and complex cardiac anomalies.

We therefore suggest that the threshold to do an echocardiogram should be low in a neonate presenting with pneumothorax, especially when there may be no acute precipitating factors such as positive pressure ventilation (PPV) being administered. The initial diagnosis of this patient was pneumothorax and associated RDS, even in the absence of PPV. The patient’s atelectasis and air trapping arose from bronchial compression mostly on the right, as is commonly seen in PAS. This infant’s PAS could have been picked up at birth if echocardiography would have been performed in the NICU. Likewise, other doctors later involved in the infant’s care should have taken into account the fact that he had wheezing that did not respond to albuterol, feeding intolerance, and overall did worse than his twin sister. These details about the child’s problems were communicated by his parents, and they were dismissed.

Our case and the mistakes that were made along the way illustrate the shortcomings of a clinical thinking mode that begins on the wrong path, with biased judgement. The tendency to rely on the first thoughts toward a diagnosis, rather than alternative, less likely explanations, is an example in the medical domain of a psychological process called the availability heuristic (Groopman 2007).

## Conclusions

The authors present the above case as a reminder to pediatric colleagues that we are on the front line of diagnosis, must remain vigilant, and when appropriate generate self-questions about the specific pieces of the clinical picture that simply do not fit. Assuming that all wheezing is some form of reactive airway disease in a pediatric patient, to diagnose the things that we already have in mind, may beget the misfortune for the patient of our having missed the very rare yet critically important disease entity.

## Abbreviations

PAS: pulmonary artery sling
NIPPV: nasal intermittent positive pressure ventilation
RSV: respiratory syncytial virus
NICU: neonatal intensive care unit
RDS: respiratory distress syndrome
LPA: left pulmonary artery
RPA: right pulmonary artery
MPA: main pulmonary artery
CPB: cardiopulmonary bypass
